# Controlled uptake of PFOA in adult specimens of *Paracentrotus lividus* and evaluation of gene expression in their gonads and embryos

**DOI:** 10.1007/s11356-022-23940-7

**Published:** 2022-11-09

**Authors:** Dario Savoca, Andrea Pace, Vincenzo Arizza, Marco Arculeo, Raffaella Melfi

**Affiliations:** Dipartimento Di Scienze E Tecnologie Biologiche, Chimiche E Farmaceutiche (STEBICEF), Università Degli Studi Di Palermo, 90100 Palermo, Italy

**Keywords:** Perfluorooctanoic acid (PFOA), Perfluoroalkylated substances (PFAS), Persistent organic pollutant (POP), Sea urchins, Embryo development, Gene expression

## Abstract

Perfluorooctanoic acid (PFOA) has been largely used in the manufacturing industry but a few years ago it turned out to be a dangerous pollutant which is now of concern for terrestrial and aquatic environments. Here, we investigated the bioaccumulation of PFOA in the sea urchin *Paracentrotus lividus* after exposure to different concentrations of the pollutant for 28 days. We observed rapid uptake of PFOA in the coelomic fluid collected weekly during the exposure period and high bioaccumulation in gonads at the end of the experiment. Interestingly, animals were also able to fast depurate when relocated to a clean environment. In addition, to assess the effect of PFOA on sea urchins’ physiological pathways, we analysed the expression profile of some marker genes both in the gonads and in the embryos obtained from parents exposed to PFOA. Our results suggest that PFOA is a persistent, bioaccumulative compound that adversely affects the health of the exposed organisms and their offspring by causing significant changes in the expression of some key target genes and the occurrence of developmental anomalies in the embryos.

**Supplementary Information:**

The online version contains supplementary material available at 10.1007/s11356-022-23940-7.

## Introduction

Perfluorooctanoic acid (PFOA) is an important representative compound of perfluoroalkylated substances (PFAS) which include a large group of over 4700 artificial chemicals (Xiao 2017; EFSA 2018) used for over 70 years for different applications in the industrial and domestic fields.

Despite restrictions on the use and production of PFAS, PFOA’s presence in the environment remains a concern for human health due to its presence in domestic and commercial products manufactured before PFAS restricted use, as well as because PFOA can be formed as a degradation product from other perfluorinated chemicals (Giesy and Kannan 2001; Betts 2007).

The widespread use of PFOA and its precursors, together with its persistence, has led to widespread environmental contamination that occurs through the processes of bioaccumulation in aquatic and terrestrial food chains (EFSA 2018; Savoca & Pace 2021).

PFOA is easily absorbed in the organisms and its elimination depends on active transport mechanisms, which vary between different species, and sexes (EFSA 2018). PFOA mainly causes organ damage (e.g. tumours) and can cause toxic effects on development and reproduction at relatively low doses in experimental animals (EFSA 2008). Besides its occurrence in environmental samples, PFOA has been often detected in humans for whom the pathways leading to PFOA exposure are still being investigated (Taniyasu et al. 2003; Kannan et al. 2004; Hu et al. 2018; Domingo & Nadal 2019; Sunderland et al. 2019). In this context, drinking water (Boone et al. 2019) and the consumption of fish and meat are among the main sources of human exposure to PFOA (Tittlemier et al. 2007; Haug et al. 2010). An important role in the release of PFOA in the environment could be attributed to municipal sewage water treatment plants (Sinclair & Kannan 2006; Sunantha & Vasuvedan 2016).

In a recent study monitoring sentinel species in coastal regions near urban activity, we recorded the presence of PFOA in *Paracentrotus lividus*, a widely distributed echinoderm with low mobility, easy management, breeding, and high resistance, ideal characteristics for bioindicator organisms (Savoca et al. 2021), or model for experimental studies (Soualili et al. 2008).

*P. lividus* is harvested in some regions of the eastern Atlantic Ocean and its gonads are appreciated as seafood in Mediterranean countries; thus, its contamination could be a direct threat to human health (Machado et al. 2019). Sea urchins can directly ingest PFOA polluted seawater or food, but another possible origin of *P. lividus* contamination is represented by the ingestion of fluorochemical products or PFOA-contaminated microplastics/fibre debris (Harada & Koizumi 2009; Gallo et al. 2018).

Besides tissues, whose analysis requires sacrificing the animals, *P. lividu*s offers the possibility to use its coelomic fluids to monitor the intake of chemical substances over time, as observed in our recent biomonitoring study (Savoca et al. 2021). In fact, several different functions, including digestion, storage, transport, and elimination of substances have been attributed to coelomocytes, circulating cells in the coelomic fluid that fills body cavities of echinoderms (Tahseen 2009). Therefore, in analogy with other echinoderms (Martín et al. 2019), it is reasonable to assume that also *P. lividus* circulating cells would be in contact with pollutants entering the organism.

In this context, while some studies have been reported on the bioaccumulation of PFOA in living organisms exposed to contaminated water (Ulhaq et al. 2015; Savoca & Pace 2021) including toxicity studies on larvae of *P. lividus* (Mhadhbi et al. 2012), there is no information on the kinetic of PFOA adsorption in adult *P. lividus* nor on the potential effects of such exposure.

Therefore, we decided to evaluate PFOA uptake and biodistribution over time in sea urchins and to evaluate changes in gene expression in the gonads of adults exposed to PFOA and in their offspring.

The PFOA uptake experiments were performed on adult *Paracentrotus lividus* farmed for a maximum of 28 days in aquariums containing different concentrations of PFOA by monitoring the presence of the fluorinated substance in the coelomic fluid as well as in gonads collected at the end of the exposure period. The expression profile of selected marker genes involved in the development, homeostasis, or detoxification was evaluated either in gonads or in late embryos generated by PFOA-exposed parents. Furthermore, the morphology of the obtained embryos was observed to assess the presence of developmental anomalies.

## Materials and methods

### Sampling and experimental design

A total of 36 adult individuals of sea urchin (*Paracentrotus lidivus*) were collected on the Northwestern coast of Sicily nearby Capo Zafferano (38°06′24″N; 13°32′14″E), the site shown to be almost not contaminated by PFOA by our previous analyses (Savoca et al. 2021), and immediately transported to the laboratory aquariums. Upon arrival in the laboratory, before housing, a sample of coelomic fluid was taken from each sea urchin in order to assess the initial PFOA concentration. Similarly, the seawater used for the experiments was analysed.

The echinoderms were acclimated for 8 days in an aquarium of 200 L of seawater filtered by 30 µm Millipore membranes (FSW). The health of the sea urchins was monitored through continuous observation and no mortality occurred during the acclimation period.

Sea urchins were then transferred for a period of 28 days in nine aquariums each filled with 15 l of FSW without (control) or with a known concentration of PFOA. The tanks were prepared in triplicate: 3 × “no-PFOA” (control), 3 × “10 ppm PFOA” (10 mg/L PFOA), and 3 × “100 ppm PFOA” (100 mg/L PFOA). PFOA stock solutions were prepared using the Perfluorooctanoic acid analytical standard (> 98% from Aldrich) and solubilised in a volume of FSW. The FSW, taken from the same sampling site of the sea urchins, was monitored weekly in each aquarium for PFOA concentration.

Four individuals of *P. lividus* have been placed in each aquarium and the colour, weight, and size (diameter without the spines measured perpendicularly to the oral-aboral axis) of each sea urchin were noted in order to recognise the individuals during the entire experimental period.

In order not to influence the uptake of the pollutant in the sea urchins, no filtering systems or feed was added to the aquariums. Salinity 35 ± 1‰, temperature 17.0 ± 1.0 °C; photoperiod 12-h:12-h light:dark and continuous aeration have been monitored daily; in addition, some physicochemical parameters (NO_2_^−^, NO_3_^−^, KH, GH, Cl_2_) were measured weekly using test strips by colourimetric method.

Each week and on day 28 or “28 + 2” (2 days after depuration), a sample of coelomic fluid (CF) was taken from each individual to evaluate the PFOA uptake.

Gonads were dissected from animals exposed to 10 ppm of PFOA on day 28 or “28 + 2,” while in the case of animals exposed to 100 ppm PFOA, sampling of the biological matrices was carried out on the nearest weekly sampling day following the onset of the strong signs of debilitation, that are reduced spine mobility, loss of spines, loss of mobility and ability to anchor to the glass of aquariums through ambulacral pedicels.

After sexual recognition of each individual sacrificed on day 28, one-third of the gonads was immediately used to recover eggs or sperm and the remaining tissue was divided into 2 tubes and stored at − 80 °C to be then used for PFOA uptake analysis or RNA extraction.

The samples, taken after the depuration, were used exclusively for chemical analyses to evaluate the pollutant elimination capacity.

### Materials, equipment, and software

LC–MS-grade methanol (from Honeywell) was used for extractions (including SPE cartridges preconditioning) and analyses. LC–MS-grade water (from PanReac Applichem) was used for HPLC–MS analyses and SPE cartridges preconditioning or washing. Ammonium acetate (from Aldrich) was used as an additive for HPLC eluents. Perfluorooctanoic acid analytical standard (> 98% from Aldrich) was used for LC–MS calibration curves and to obtain spiking solutions which were freshly prepared and checked for their PFOA content before each batch of analyses. SPE cartridges Strata TM-X-AW (33-µm polymeric weak anion 200 mg/6 mL tubes) were purchased from Phenomenex and used for PFOA extraction from seawater samples. PFOA-free polypropylene micropipette tips were used for quantitative small volume withdrawals. In order to prevent any type of PFOA contamination, the equipment used for sampling and extraction procedures was washed with the same methanol used for extraction and analysis. LC–MS analyses were performed using a 6540 UHD Accurate-Mass Q-TOF LC/MS (Agilent Technologies) equipped with a Dual AJS ESI source. Histograms, tabs, and statistical analysis have been realised using Excel 2016 (Microsoft) and PAST3 (Hammer et al. 2001).

### PFOA extraction and analysis

The PFOA analytical and extraction processes were adapted to the type of matrix to be analysed following the same methods, procedures and instruments described by Savoca et al (2021) as well as the calculation of recovery percentages (*R* %), that were checked per each batch of analyses with slight modifications.

0.3 mL of the coelomic fluid was taken from all collected specimens by inserting the needle of a 1-mL syringe through the peristomal membrane (Arizza et al. 2013). The extraction of PFOA from the coelomic fluid samples was performed without prior separation of the coelomic fluid from the coelomocytes.

After PFOA extraction, seawater, coelomic fluid, and gonads samples collected from aquariums containing 10 ppm or 100 ppm of PFOA were diluted in LC–MS grade methanol at a ratio of 1:10 or 1:100 respectively. In the case of samples, collected from control tanks, where PFOA was not detected, analyses were repeated for confirmation on concentrated sample extracts.

### In vitro fertilisation and embryo observation

Egg samples were collected from dissected gonads that were dropped in 30 mL of filtered seawater (FSW). Eggs were released, separated from ovaries by filtering through a silk mesh, and washed twice with fresh FSW. After each wash, eggs were allowed to settle by gravity and re-suspended in 250-mL FSW for fertilisation. Male gonads were placed into 10-mL tubes and sperm was kept dry on ice until use.

Just before fertilisation, 25 μL of dry sperm were diluted in 5 mL of FSW, and a drop was added to the suspension of eggs. Fertilisation was followed by light microscopy and allowed to proceed for 5 min before washing the eggs twice with FSW. All the procedures were performed with lab glassware and stainless steel tools, avoiding the use of plastic. Embryos were kept in FSW at a constant temperature of 18 °C for 48 h post fertilisation (p.f.) with gentle mechanical stirring, allowed to grow up to the stage of early pluteus, and collected for further analyses. Before morphological observations, swimming larvae were fixed with 5 µL of 37% formaldehyde in 20 mL of seawater and immediately photographed (Leica DM IL microscopes associated with a Leica DFC 320 camera, using × 10, × 20, × 40 magnifications). Alterations of larvae were evaluated using a three-level classification according to Carballeira et al. (2012): (level 1) incorrect location of skeletal rods; (level 2) incomplete or absent skeletal rods; (level 3) blocked development.

### Gene expression analyses

Gonads and embryos obtained from PFOA-exposed and control sea urchins were collected and stored at − 80 °C for gene expression analyses. Total RNAs were extracted from the samples by a commercial kit (PureLink RNA mini kit, Invitrogen) and, following quantification (NanoDrop ND-1000 UV–Vis Spectrophotometer and 1% Agarose/1X TBE gel migration) and removal of the genomic DNA (RQ1 RNase-Free Dnase, Promega), 1 µg per sample was reverse-transcribed (High-Capacity cDNA Reverse Transcription Kit, Applied Biosystems).

Gene expression by quantitative real-time polymerase chain reaction (qPCR) was performed with the ABIPRISM 7500 system (Applied Biosystems, Forster City, USA) and Power Sybr Green PCR Master Mix (Applied Biosystems, Forster City, USA) as indicated by the manufacturer. Each reaction was performed in triplicate and as an internal reference gene, we used 18S ribosomal RNA.

We analysed in gonads and embryos: MDR1 (multi drug resistance protein 1), a marker of detoxification and survival; Blimp1 (PR / SET Domain) and DNMTs (DNA methyltransferase), transcriptional and post-transcriptional regulators of gene expression.

Only in gonads: HSP70 (Heat shock protein 70), a marker gene of the stress response; HIF1 (hypoxia-inducible factor 1-alpha transcription factor), linked to detoxification and survival; P38 MAPK (p38 mitogen-activated protein kinases), a marker of transcription and post-transcription regulation.

Only in embryos: SM30 and SM50 genes, coding spicule matrix proteins, involved in skeletal morphogenesis.

All genes and oligonucleotide primers used in this study were already described in other papers (Ragusa et al. 2013; Migliaccio et al. 2014; Ruocco et al. 2016; Ragusa et al. 2017a, b; Di Natale et al. 2019; Masullo et al 2021). Data were analysed through the comparative threshold cycle (CT) method (Schmittgen & Livak 2008).

Amplification conditions were: initial denaturation and enzyme activation 10 min at 95° C, followed by 40 cycles each consisting of 15 s at 95 °C and 60 s at 60 °C. At the end of the amplification, a melting curve analysis was run (from 65 to 95 °C). Variation of expression levels was calculated as relative expression ratios of the analysed genes concerning control embryos.

**Fig. 1:**
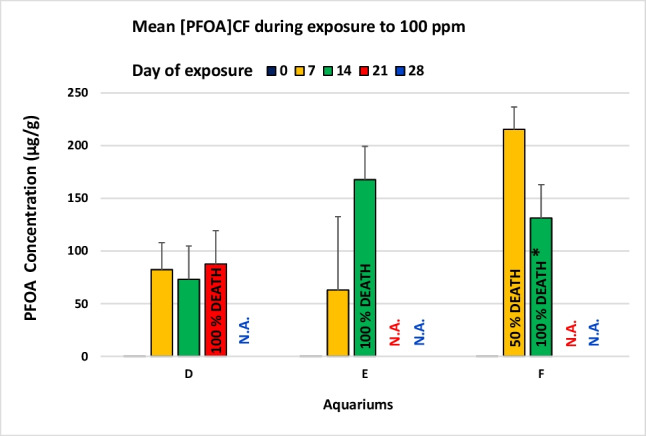
Bar charts show the mean of [PFOA]_CF_, measured weekly (days 0, 7, 14, 21, 28), in individuals belonging to the same tank (D, E, or F) added with 100 ppm PFOA. N.A = not available. *The average [PFOA]_CF_ measured on day 14 in aquarium F refers only to two specimens (F1 and F2). Data are expressed as arithmetic mean ± standard deviation (SD) of the performed replications

**Fig. 2:**
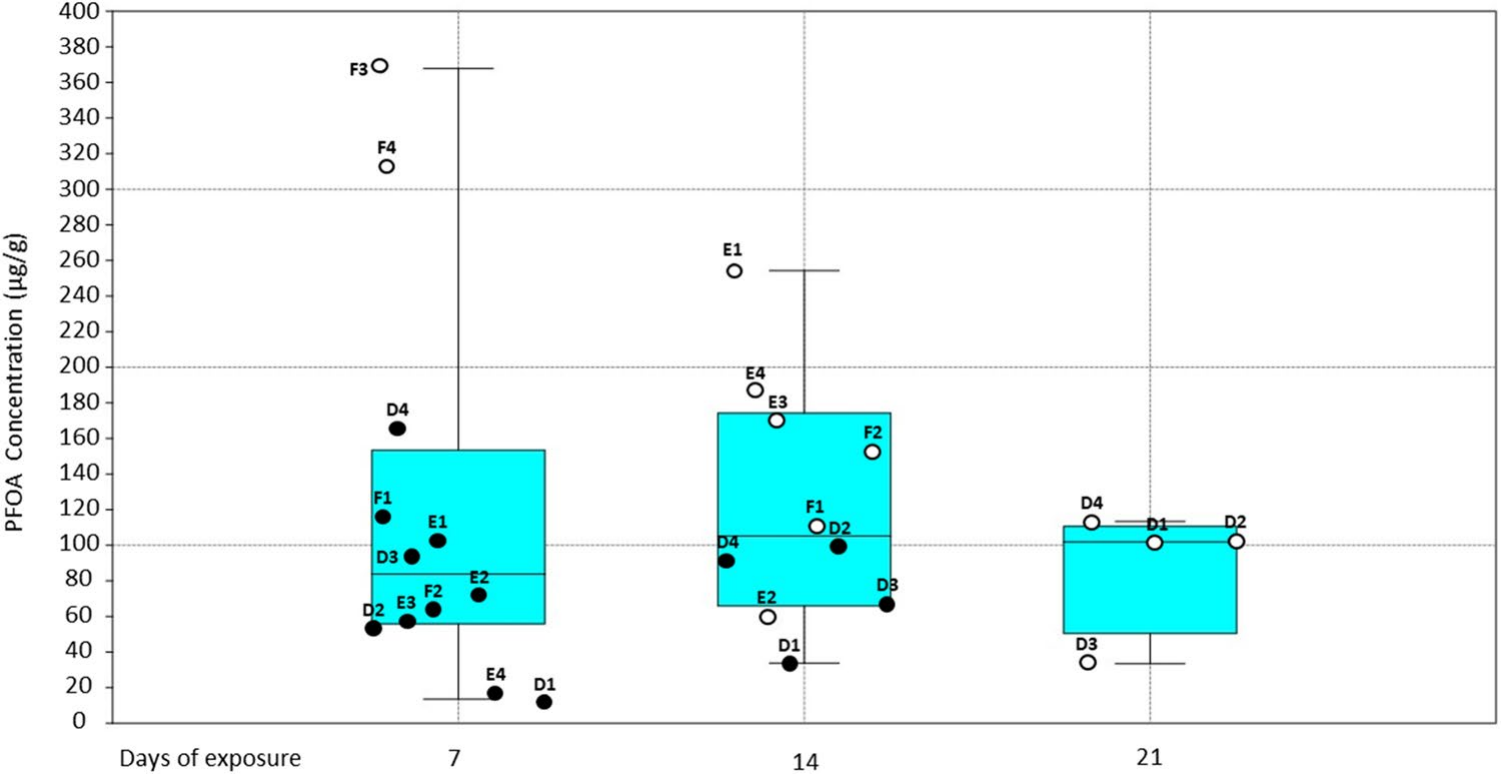
Distribution of [PFOA]_CF_ in each sea urchins exposed to 100 ppm where the 25–75 percentiles are drawn using a box; minimum and maximum are shown at the end of the thin lines (whiskers), while the median is marked as a horizontal line in the box fitting. Empty dots indicate unhealthy/dissected individuals and full ones healthy individuals. In the x-axis, the days of sampling of the coelomic fluid are shown (the spreading of dots along the x-axis within the same group of sampling is arbitrary chosen to avoid overlapping of points), in the y-axis the measured concentration is expressed in ppm

2The results of gene expression are presented in box plots showing the fold change value 2 ^–ΔΔCT^ (Figs. 1 and 2).

### Statistical analyses

**Fig. 3:**
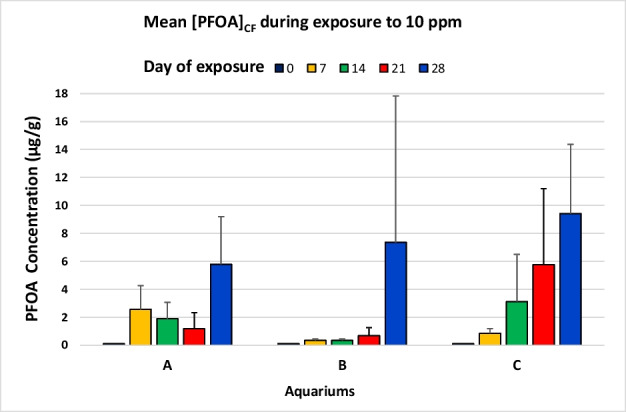
Bar chart shows the mean of [PFOA]_CF_ measured weekly (days 0, 7, 14, 21, 28) on individuals belonging to the same tank (A, B, or C) containing 10 ppm of PFOA. Data are expressed as arithmetic mean ± standard deviation (SD) of the performed replications

Box and jitter graph (Fig. 2), Shapiro–Wilk’s test, Levene’s test, one-way ANOVA, and Kruskal–Wallis test were performed using PAST software 3.25 (Hammer et al. 2001) with a significance level of 0.05, while bar charts (Figs. 1 and 3) have been realised using Excel 2016 (Microsoft).

One-way ANOVA was performed after testing the normality of the distribution and the homogeneity of the variance with Levene’s test. In the case of normality test failure or heteroscedasticity, it was applied the non-parametric Kruskal–Wallis analysis of variance (Wayne 2005).

ANOVA or Kruskal–Wallis were used to compare the following: (i) the embryos obtained from control vs treated adults, (ii) PFOA concentration of groups of sea urchins, (iii) and for gene expression analyses to determine differences between treated groups and respective control groups.

For gene expression analyses, statistical significance for each target gene among triplicates Cq values of control and the treated sample is shown as asterisks (*: *p* < 0.05); relative mRNA levels are shown as mean ± SD (*n* = 3).

## Results

### Chemical analyses of water, coelomic fluid, and gonads

Seawater samples were taken each week and checked for the presence of PFOA by solid-phase extraction (SPE) followed by LC–MS analysis (Hong et al. 2015). The percentage of PFOA recovery from seawater was 120% and the average of the PFOA concentration levels in all the aquariums, before the addition of the pollutant, was less than 10 ppt (ng/L) as it was in the control aquariums (X, Y, Z). In all aquariums, PFOA concentration was stable and close to the nominal one during all the experimental periods.

Concentrations of PFOA in the coelomic fluid before the beginning of the experiment were below 15 ppb (μg/L). The percentage of PFOA recovery from CF was 70%.

Furthermore, as expected, [PFOA]_CF_ in sea urchins in the control aquariums, after weekly monitoring for 28 days, never showed particular variations, always remaining in the order of a few ppb (Fig. S1 in supporting information) and in the gonads, the presence of PFOA has never been detected (Tab. S1 in supporting information).

On the other hand, chemical analysis of the [PFOA]_CF_ in sea urchins exposed to 10 and 100 ppm showed that they bioaccumulated the pollutant in during the exposure period.

In the 100 ppm experiment, no significant differences in averages were recorded between the contamination profiles of the coelomic fluid in the three aquariums for each of the three monitoring samplings: day 0 (Kruskal–Wallis test: *p* = 0.156), day 7 (Kruskal–Wallis test: *p* = 0.174), day 14 (ANOVA: *p* = 0.132) (Fig. 1).

When considering individuals, among sea urchins exposed to 100 ppm, two (F3 and F4) showed the signs of health decay in the first week, and all those in tanks E and F followed the same fate from day 7 to 14, while sea urchins in tank D (D1-D4) could not survive longer than 21 days. At the end of each week (days 7, 14, and 21) all the animals appearing agonizing were dissected to collect the matrices to be analysed. [PFOA]_CF_ was determined, and values are reported in Fig. 1. We observed that from the first week all the animals could uptake PFOA but showed a different rate of absorption (Fig. 2 and Fig. S2).

Indeed, the amount of PFOA measured on day 7, day 14, and day 21 displayed that the accumulation tendencies varied between the different individuals exposed to the same concentration of PFOA (Table 1).

**Table 1:** Summary statistics sea urchins and [PFOA]_CF_ exposed to 100 ppm. Concentration values are expressed in µg/g

	Number of individuals	Shapiro–Wilk *p*-value	Mean	Standard deviation	Median	Range (Min–Max)
Day 7	12	1.06 · 10^−2^	120.137	111.484	83.760	13.550–367.916
Day 14	10	0.73	122.670	67.490	105.251	33.800–254.232
Day 21	4	4.64 · 10^−2^	87.650	36.512	101.894	33.488–113.323

The PFOA contamination profiles of the coelomic fluid samples of sea urchins belonging to the three aquariums with a nominal 10 ppm PFOA concentration, were different in averages in the first two weekly monitoring sampling: day 0 (Kruskal–Wallis test: *p* = 0.053), day 7 (Kruskal–Wallis test: *p* = 7 · 10^–^3), day 14 (Kruskal–Wallis test: *p* = 0.025), day 21 (Kruskal–Wallis test: *p* = 0.389), day 28 (ANOVA unidirectional: *p* = 0.819) (Fig. 3).

Differently from the exposure to 100 ppm, sea urchins in tanks A, B, and C, where nominal PFOA concentration was 10 ppm, appeared healthy until the end of the experiment (28th day) when the maximum pollutant concentration was recorded in all samples (Fig. 3 and Fig. S3).

Also in this case, we recorded a high variability in the rate of uptake of single individuals at each weekly monitoring sampling (Table 2).

**Table 2:** Summary statistics sea urchins and [PFOA]_CF_ exposed to 10 ppm. Concentration values are expressed in µg/g

	Number of individuals	Shapiro–Wilk *p*-value	Mean	Standard deviation	Median	Range (Min–Max)
Day 7	12	4.26 · 10^−4^	1.257	1.440	0.852	0.262–5.454
Day 14	12	1.85· 10^−4^	1.785	2.457	0.949	0.266–8.930
Day 21	12	1.97· 10^−4^	2.544	4.129	0.687	0–13.260
Day 28	12	3.68 · 10^–2^	7.520	5.544	7.448	0.475–25.431

To verify the biodistribution of the pollutant in organs involved in possible transfer to the offspring, the concentration of PFOA in gonads [PFOA]_G_ was analysed on day 28 (Table 3), or at day “28 + 2” after depuration (Tab. S2 in supporting information), since it is necessary to sacrifice the animals to collect this tissue. The percentage of PFOA recovery from gonads was 73%.

**Table 3:** PFOA concentration (ppm) in the coelomic fluid (CF) and gonads (G) of individuals exposed to 10 or 100 ppm on the day of death (7, 14, 21, or 28) when animals were dissected for matrices collection. *M* male; *F* female; *U* undetermined

Individuals exposed to 10 ppm	Day of death	[PFOA]_CF_ (µg/g)	[PFOA]_G_ (µg/g)
A1 (F)	28	5.66	4.56
A2 (M)	28	9.96	
A3 (F)	28	0.57	4.41
A4 (U)	28	6.99	
B1 (F)	28	25.43	
B2 (M)	28	0.41	40.54
B3 (U)	28	2.89	
B4 (M)	28	0.67	11.39
C1 (M)	28	10.08	13.02
C2 (F)	28	17.25	
C3 (U)	28	5.43	
C4 (F)	28	4.90	8.80
Individuals exposed to 100 ppm			
D1	21	102.55	511.06
D2	21	101.24	609.38
D3	21	33.49	354.25
D4	21	113.32	497.81
E1	14	254.23	346.37
E2	14	60.04	370.42
E3	14	170.24	277.22
E4	14	186.32	661.36
F1	14	110.00	464.58
F2	14	152.62	223.57
F3	7	367.92	388.28
F4	7	312.24	884.14

Sea urchins of aquariums D, E, and F exposed to 100 ppm PFOA, which appeared dying, and thus dissected one or more weeks before the end of the experiment, showed much higher [PFOA]_G_ values than those exposed to 10 ppm PFOA for 28 days (see Table 3).

We also detected that PFOA concentration values in gonads of treated animals (either with 10 or 100 ppm PFOA) were generally relatively higher (with only one exception, A1 in Table 3) than those found in the CF taken the same day from the same individual (Kruskal–Wallis test: *p* = 0.078 for 10 ppm experiment and ANOVA: *p* = 5.59 · 10^−5^ for 100 ppm experiment) (Table 3).

Furthermore, at the end of the 28 days, it was possible to identify the sex of 6 individuals exposed to 10 ppm PFOA and from the chemical analyses it was observed that the concentrations of PFOA in the gonads in the 3 males on average (21.65 ppm) were significantly higher than that found in the 3 females (5.92 ppm) (Kruskal–Wallis test: *p* = 0.049).

After 28 days of exposure to 10 ppm of PFOA, the decontamination of the coelomic fluid was verified by moving six of the twelve PFOA-exposed individuals (2 from each tank A, B, or C) in an aquarium with uncontaminated FSW and analysing both coelomic fluid and gonads after 2 days of depuration (28 + 2) (Table S2 in supporting information).

The decontamination was calculated as a percentage difference:

% decontamination = 100 · (1-[PFOA]_CF_^28+2^/[PFOA]_CF_^28^) and results showed average decontamination of coelomic fluid of 87%.

### Morphological observation of embryos

The presence of developmental alterations was investigated in embryos obtained from the in vitro fertilisation of gametes from *P. lividus* specimens exposed to 10 ppm PFOA and sacrificed on day 28. Eggs were isolated from mature gonads of three females from aquariums A, B, and C, and of three females from control aquariums X, Y, and Z, and were fertilised with sperms from males belonging to the same treatment or control aquarium, respectively.

**Fig. 4:**
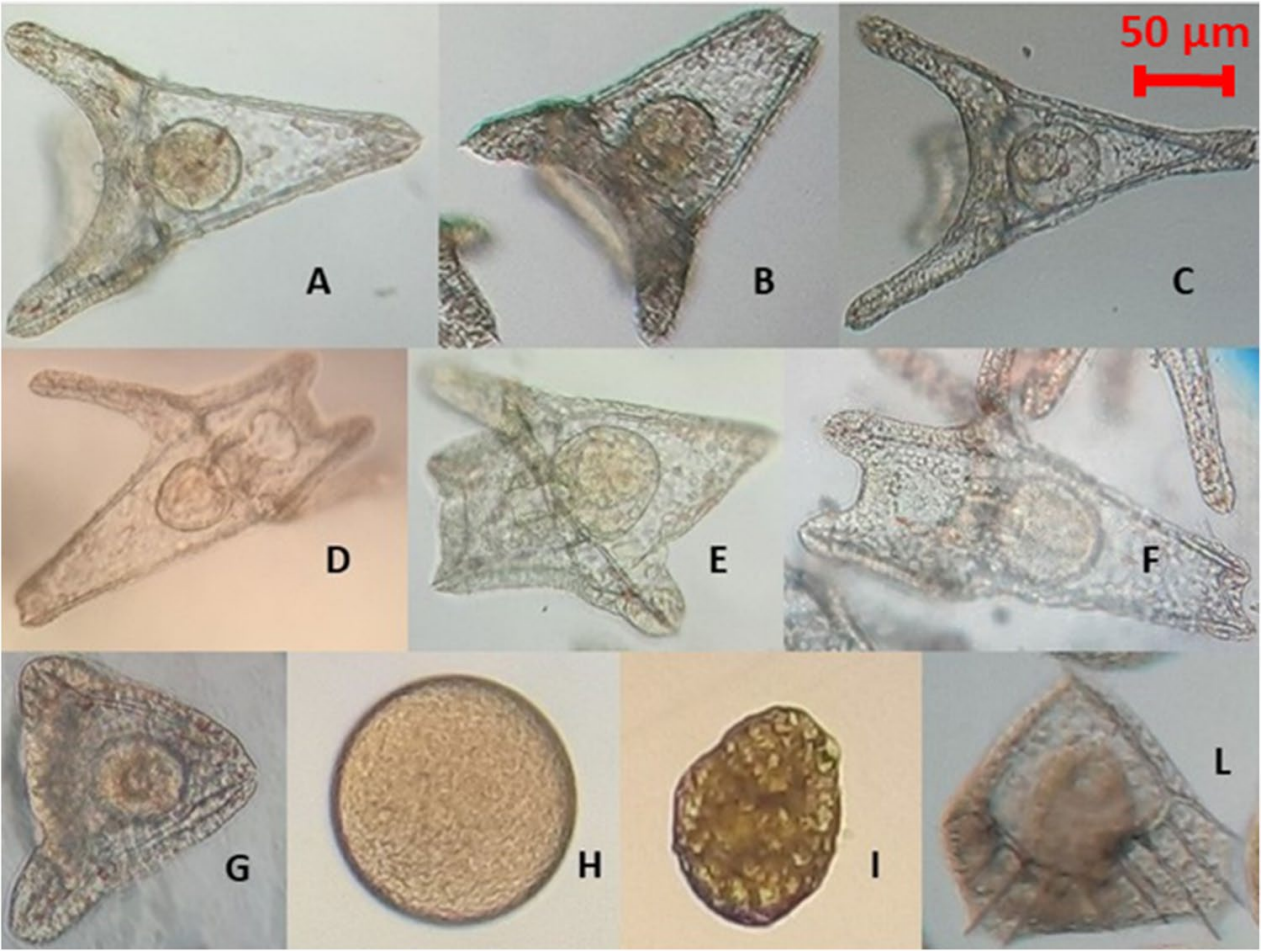
Representative images of embryos obtained from parents exposed to 10 ppm PFOA and controls. A: normal pluteus; B: level 1-separated tips; C: level 1-crossed tips; D-E: level 1-folded arms, extraskeletal rods; F: level 2-fractured ectoderm; G: level 3-blocked development. Unclassified levels: H: unfertilised egg; I: degraded egg/embryo; L: aberrant development, nonspecific malformation

Fertilisation success was greater than 98% in all sets of eggs and the developmental stages were followed but only counted and observed carefully at the pluteus stage (48 h p.f.). Each fertilisation was performed in triplicate and 300 embryos per batch were counted and morphologically analysed. Thus, to find out if the parent’s PFOA exposure could lead to developmental defects in the progeny, 900 embryos for each of the 6 cases were observed by light microscopy (Fig. 4 and Fig. S4).

**Fig. 5:**
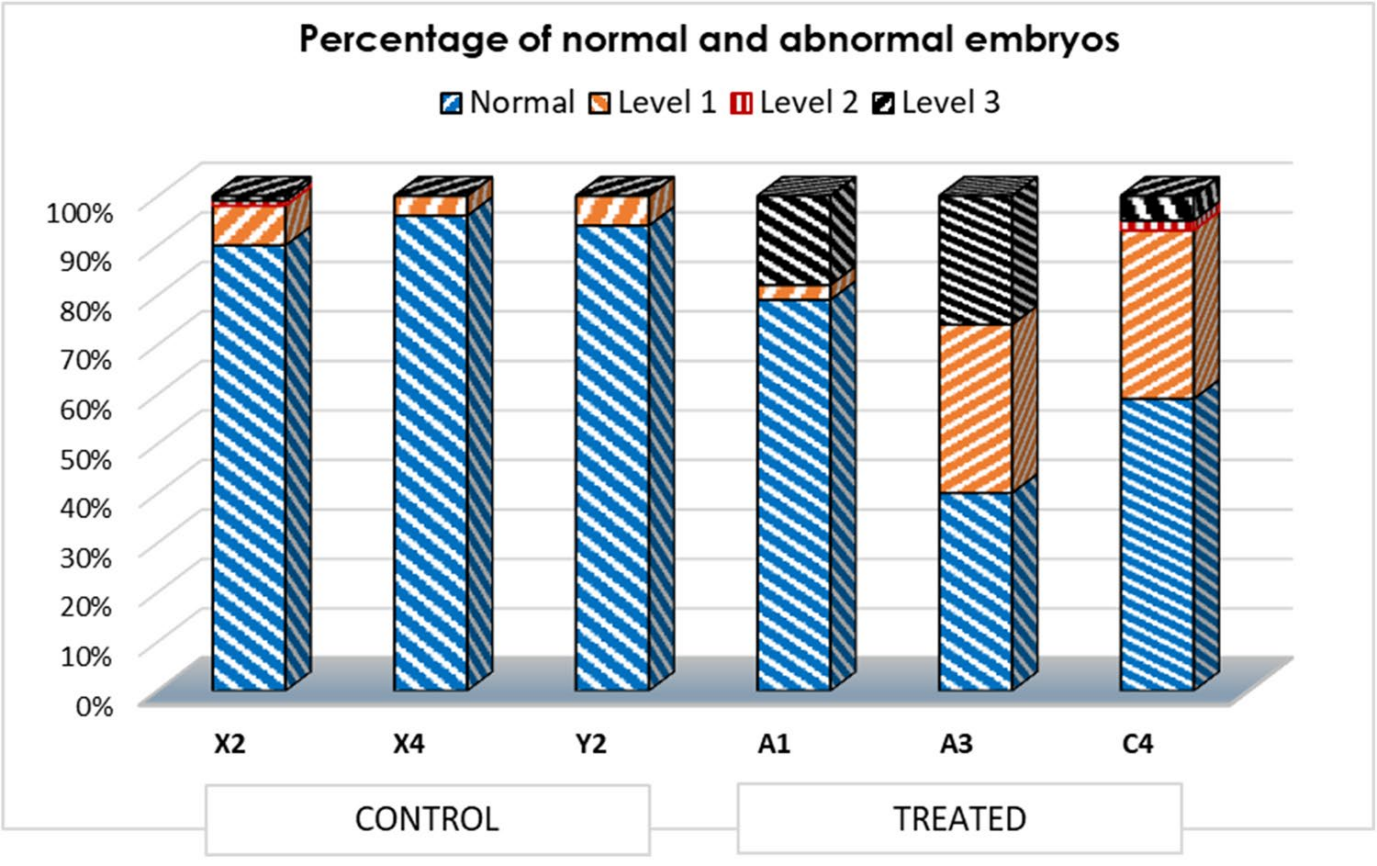
Panel of anomalies percentage, reported for each level of severity (level 1, 2, 3), observed in embryos at 48 h p.f. X2, X4, Y2: embryos obtained from sea urchins not exposed to the pollutant; A1, A3, C4: embryos obtained from sea urchins exposed to 10 ppm PFOA

The percentage of anomalies reported for each level of severity was calculated after observing the 900 embryos obtained from the gonads of each female sea urchin (Fig. 5). Unfertilised eggs, degraded embryos, and nonspecific malformations were not considered in observations because they were less than 2% in all observed groups.

When comparing the number of larvae with developmental defects in the progeny of treated animals with that of controls, we found up to 60% of defects in the former (e.g. A3 in Fig. 5) but only up to 10% in the latter. ANOVA showed that the difference in the percentage of normal embryos obtained from treated and untreated parents was significant (*p* = 0.041).

Nevertheless, in order to further investigate the anomalies of all embryo groups, we also considered the different severity of level 1 malformation: low grade (L), medium grade (M), high grade (H) (Fig. S5). This analysis shows a significant difference for the three grades of level 1 severity: (ANOVA: *p* = 6·10^–3^ for a low grade; ANOVA: *p* = 0.025 for a medium grade; Kruskal–Wallis test: *p* = 0.046 for high grade, highlighted a major frequency of higher grades of severity in embryos obtained from sea urchins exposed to PFOA compared to control groups (Fig. S6).

### The effects of PFOA on Gene expression

In order to assess the effects of 28 days of exposure to 10 ppm PFOA on pathways of survival, detoxification, or development in treated sea urchin adults and their progeny, we analysed in gonads and embryos the gene expression profiles of selected marker genes linked to those mechanisms.

#### Gene expression profile in gonads

We analysed the gonadal gene expression profile of six different marker genes. Gene expression has undergone variations in function of the accumulation of the pollutant for each individual, showing, in general, overexpression of the target genes for uptake levels higher than 10 ppm and an underexpression for bioaccumulation values lower than 10 ppm.

**Fig. 6:**
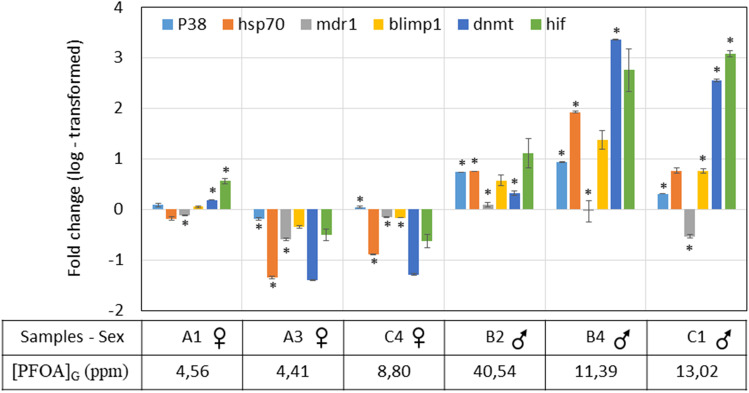
Graph representation showing differences in the expression level of each analysed gene in *P. lividus* gonads at day 28; y-axis: the scale of the logarithmic values of the fold change; x-axis: samples codes. Bars represent the mean values from the three different technical replicates ± SD. Statistical significance for each target gene among triplicates Cq values of control and the treated sample is shown as asterisks (*: *p* < 0.05)

The two genes involved in canonical stress response mechanisms, P38 MAPK and HSP70, were up or downregulated, with different intensities, based on the sex of the individuals. In fact, P38 MAPK was upregulated in all the gonads, except the female A3, but with higher values in males. Similarly, HSP70 was upregulated in the three male gonads (B2, B4, and C1) but downregulated in females (A1, A3, and C4). Of the two genes involved in detoxification and survival mechanisms, MDR1 and HIF1A, the first was downregulated in 5 out of 6 samples (slightly upregulated only in B2), while the second gene, again, was upregulated in the three male gonads but downregulated in females except for A1. In the same way, the genes involved in transcriptional regulation, BLIMP1 and DNMT1 are both upregulated in males and downregulated in two out of three female gonads (A3 and C4). In A1, they are both just very weakly upregulated (Fig. 6).

#### Gene expression profile in embryos

**Fig. 7:**
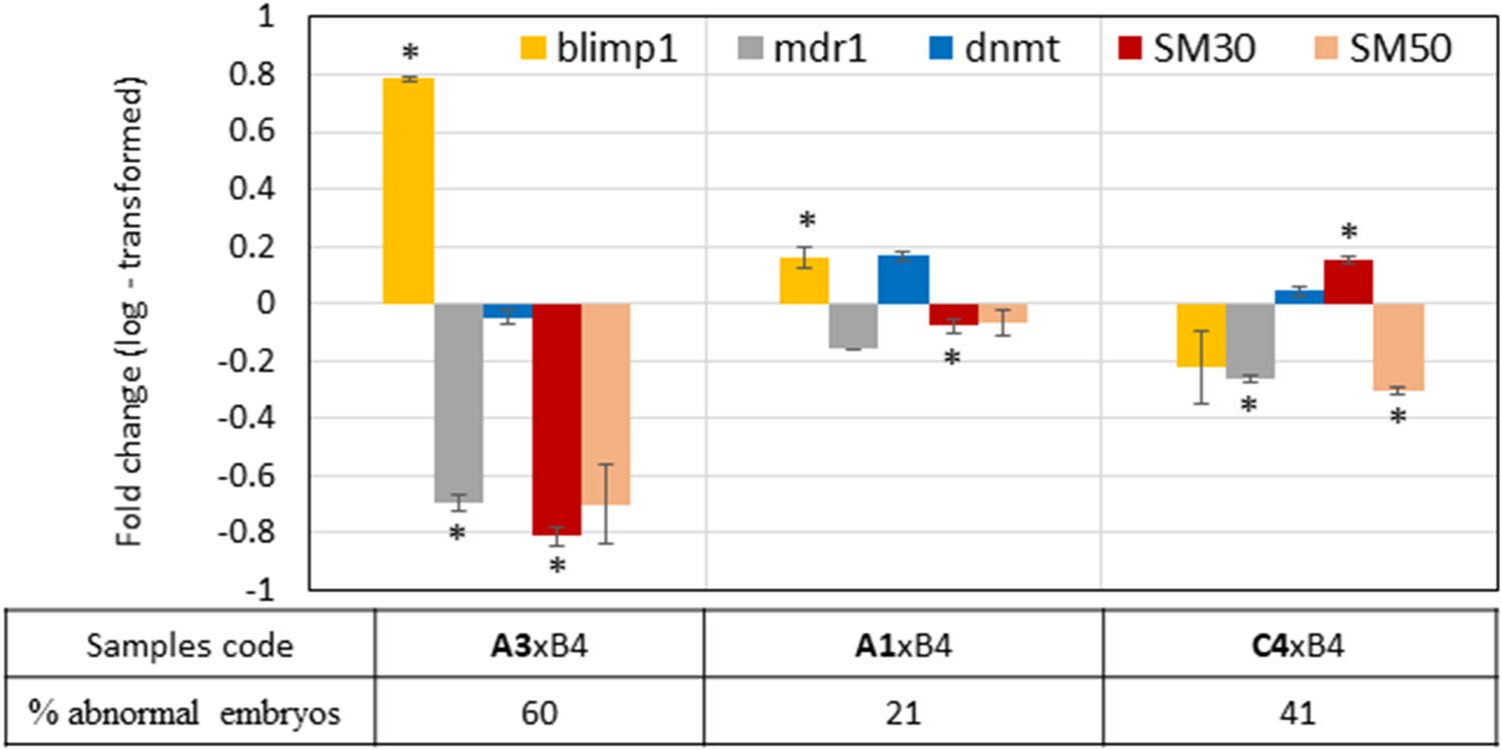
Graph representation showing a difference in the expression level of target genes compared to control embryos, normalised with 18S expression. y-axis: the scale of the logarithmic values of the fold change, x-axis: embryo samples codes (A1xB4, A3xB4, C4xB4) and the percentage of abnormal embryos. Bars represent the mean values from the three different technical replicates ± SD. Statistical significance for each target gene among triplicates Cq values of control and the treated sample is shown as asterisks (*: *p* < 0.05)

Alterations in gene expression patterns in the embryos obtained from couples of parents exposed to 10 ppm of PFOA are reported in Fig. 7. Compared to changes in gene expression recorded in the gonads, those recorded in embryos were less significant. BLIMP1 was upregulated in **A1**xB4 and **A3**xB4 and downregulated in **C**4xB4, MDR1 was downregulated in the three analysed samples and DNMTs were almost unaffected but just slightly upregulated only in **A1**xB4. As regards the two embryos’ specific genes involved in sea urchin larval skeletal morphogenesis, SM30 and, SM50, qRT-PCR revealed that in all embryos SM50 was downregulated, while SM30 was downregulated in **A1**xB4 and **A3**xB4 and upregulated in **C4**xB4.

## Discussion

This study provides the first contamination data and related effects of exposure to PFOA in the sea urchin *P. lividus* and its progeny. We provide evidence that this pollutant can be bioaccumulated in the coelomic fluid and in the gonads of adults and that PFOA exposure can perturb the gonadal expression of genes involved in detoxification pathways. Furthermore, we highlight the possible transmission of parental stress to the offspring at morphological but also molecular level.

Chemical analysis of the [PFOA]_CF_ in sea urchins exposed to 10 ppm compared to those exposed to 100 ppm showed a significant difference in dose-dependent bioaccumulation of the pollutant for each of the three samplings: day 7 (ANOVA: *p* = 1.27 · 10^−3^), day 14 (ANOVA: *p* = 4.38 · 10^−6^), day 21 (ANOVA: *p* = 6.49 · 10^−7^). Indeed, different effects on animal health were recorded between the two groups, in fact, unlike the group exposed to 10 ppm of PFOA, where sea urchins appeared healthy until day 28, sea urchins exposed to 100 ppm showed severe symptoms of debilitation all along and at the end of the experimental period (see Fig. S7 in support information).

We also noticed that individuals F3 and F4 exposed to 100 ppm, dissected at the end of the first week, when they appeared irreversibly compromised, the [PFOA]_CF_ was statistically significantly higher (ANOVA: *p* = 1·10^−4^) than in healthy individuals, in the same day, and at least three times higher than in those of the same group but dissected at the end of the third week. We hypothesize that the decline of sea urchin health at different times and before the end of the experiment of exposure at the highest PFOA concentration (100 ppm) could be due to either a higher rate of absorption (tank E and F) or a longer period of exposure (tank D) and thus to the different capacity among individuals to accumulate the pollutant, each showing different values of [PFOA]_CF_ (See Fig. S2 in supporting information) and of [PFOA]_G_ the same sampling day.

Among sea urchins exposed to 10 ppm, the uptake trend was different in the different tanks (Fig. 3), in different individuals and even within the same subjects over time (See Fig. S3 in supporting information) probably according to the intraspecific transport mechanisms of PFOA (assimilation and elimination) mediated also by the coelomocytes that play a key role in the defence reactions and important immune functions, such as endocytosis and exocytosis (Smith et al. 2010).

Importantly, we observed that *P. lividus* specimens can quickly uptake the pollutant in the coelomic fluid and gonads as well as quickly activate a decontamination process that significantly eliminates the PFOA after exposure to a clean environment (depuration). In fact, after 2 days of depuration in an aquarium with FSW without pollutants, PFOA reduction in the coelomic fluid was ranging between 62 and 99% for samples initially exposed to 10 ppm PFOA (Table S2 in supporting information). It is interesting to note that in four subjects exposed to 100 ppm PFOA, between days 7 and 14 (D3, D4, F1, E2), and in one of tank D (D3), between days 7 and 21, [PFOA]_CF_ decreased (Fig. S2 in Supporting Information), most probably because of the activation of such detoxification mechanisms.

Analysing the data relating to the levels of PFOA in the coelomic fluid before and after the depuration (in the same individuals) and those of the gonads before and after the depuration (in different individuals), we can hypothesize that the coelomic fluid undergoes decontamination at a greater rate than the gonads.

With the aim to observe any developmental defect in the offspring of treated parents, we performed in vitro fertilisation with gametes obtained from parents both exposed to 10 ppm PFOA, while control embryos derived from gametes obtained from untreated (control) parents. Both normal and severely affected larvae were found in each batch of treated or control embryos (see Fig. 5). However, the embryos obtained from sea urchins exposed to the pollutant had a significantly higher incidence of malformations and severity. The uneven distribution of severity levels could be due to high variability in the recovery capacity of individual embryos, as we observed for adults. This result represents the first evidence of possible morphological effects on the progeny of sea urchins exposed to PFOA and is in agreement with a previous report by Mhadhbi et al. (2012) where the pollutant was directly administered to the embryos.

Gene expression reprogramming may help sea urchins to adapt to stress, thus the changes in gonadal gene expression that we observed after analysing 6 different marker genes, strongly suggest that in sea urchins PFOA activates damage mitigating pathways that respond rapidly to external stress, in order to maintain cellular homeostasis. P38 MAPK, which plays an essential role in regulating cellular defence mechanisms of the stress response (Park et al. 2020) and HSP70, a class of molecular chaperones that participate in stress response and environmental acclimatisation (Zuo et al. 2017), were both affected by PFOA and, in both cases, some correlation with sex and PFOA absorption can be hypothesised. Similarly to our results, P38 upregulation was observed in some crabs exposed to other POPs (Park et al. 2020) and its upregulation as a function of PFAS concentration was observed in frog embryos exposed to PFOS (San-Segundo et al. 2016). The same trend of up or downregulation linked to sex and absorption was noticed in the gonads also for MDR1, a key component in defence systems induced by various types of oxidative stress associated with exposure to xenobiotics (Gökirmak et al. 2012), and HIF1A, a gene involved in the regulation of the cellular and systemic homeostatic response to hypoxia (Majmundar et al. 2010). The genes involved in transcriptional regulation, BLIMP1, which plays a key role in the processes leading to the specification of tissues and determination of the germline (Ohinata et al. 2005), and DNMT1, which is responsible for maintaining DNA methylation patterns (Schulz et al. 2018), are both up regulated in males and mainly downregulated in female gonads. This could be expected since it is known that exposure to toxic substances affects DNA methyltransferase expression, altering the complex methylation pattern (Di Natale et al. 2019) and it was already published that, in the sea urchin *Glyptocidaris crenularis*, the perfluorooctane sulfonate (PFOS) induced DNA methylation changes (Ding et al. 2015). It is worth noting that gonadal DNMTs altered expression may affect chromatin accessibility and can be responsible for the creation of a heritable epigenetic chromatin state.

This study also provides the first insights into the toxic effect of PFOA in the offspring of exposed parents at the molecular level. We found that in the embryos obtained from couples of parents exposed to 10 ppm of PFOA (Fig. 7) both MDR1 and BLIMP1 gene expression profiles differed from controls, as it may be expected if considering their involvement in detoxification function. In particular, MDR1 was downregulated in all three batches, as it was already observed after exposure to other substances that induce malformations in *P. lividus* embryos, where it activates different defence mechanisms (Ruocco et al. 2016). DNMTs, mostly unaffected, showed a mild downregulation only in the embryos A3xB4 in which the largest number of total anomalies were detected (60%).

In the case of SM50 which plays a role in defining the extracellular space where spicule deposition occurs, and SM30, which plays a role in the secretion of spicule components (Urry et al. 2000), differences from the expression in control embryos could explain the percentage of malformations linked to spicules shape and number in the offspring of treated parents. This general trend of downregulation of the embryo’s specific genes involved in skeletogenesis is in line with other studies where pollutants induced abnormalities in skeletal morphogenesis of sea urchins’ larvae (Duloquin et al. 2007; Marrone et al. 2012; Varrella et al. 2014). More generally, it is interesting to note that the magnitude of the change in expression of all the genes, excluding DNMT, reflects the percentage of morphological abnormalities.

## Conclusion

Our results showed that the absorption of PFOA in sea urchins is evident from the first week of exposure to the pollutant and that during the period of exposure the levels of pollutants varied over time in the same and different individuals.

This variability could be attributed to different immunophysiological mechanisms (probably mediated also by coelomocytes) and to the different sexes of sea urchins.

In this context, it is possible to hypothesize that during the exposure period *P. lividus* activate self-defence mechanisms, generally activated by adverse external conditions, to protect themselves from the negative effects of PFOA. Indeed, in the gonads of treated animals, we observed that genes involved in detoxification, stress defence and key factors of the general transcriptional regulation are all affected by PFOA exposures.

Importantly, we also demonstrated that *P. lividus*, at least when exposed to 10 ppm PFOA for 28 days, is able to depurate when relocated from a contaminated to an uncontaminated environment, thus making this species potentially resilient to sporadic contamination conditions. Defence mechanisms lead to the elimination of PFOA but are probably limited by a saturation/accumulation threshold that could irreversibly affect the animals, as suggested by the outcome of the 100 ppm exposure experiment.

This study suggests for the first time that PFOA parental exposure may affect the morphology of the progeny and may induce in the embryos the reprogramming of the expression pattern of general transcriptional regulators, like BLIP1, and of developmental key genes, like SM30 and SM50, which may be, at least in part, responsible for the skeletal anomalies that we observed. Thus, the effects of parental exposure may be reflected in the next generation, influencing the development processes.

The present study highlights that, unlike the coelomic fluid, which showed rapid bioaccumulation of PFOA as well as rapid purification, the gonads do not seem to purify so quickly (suggesting that great care must be taken when these animals are destined directly for human consumption). In this context, PFOA levels in the coelomic fluid would represent the instantaneous indicator of its presence in the environment, while the gonads would give a picture of the contamination reflecting a wider pollution period. Thus, the sea urchin could be considered a PFOA bioindicator in a marine ecosystem.

## Supplementary Information

Below is the link to the electronic supplementary material.

Supplementary file1 (DOCX 736 KB)
