# Supplementary material for: Controlled uptake of PFOA in adult specimens of Paracentrotus lividus and evaluation of gene expression in their gonads and embryos
Source: Environ Sci Pollut Res Int. 2022 Nov 9;30(10):26094–106. doi: 10.1007/s11356-022-23940-7 (PMC9995410; doi:10.1007/s11356-022-23940-7)
Supplement: Supplementary file 1 — Supplementary file1 (DOCX 736 KB) [file 11356_2022_23940_MOESM1_ESM.docx]

**Supporting information:**

**Controlled uptake of PFOA in adult specimens of *Paracentrotus lividus* and evaluation of gene expression in their gonads and embryos**

*Dario Savoca*, Andrea Pace, Vincenzo Arizza, Marco Arculeo, and* *Raffaella Melfi*

Dipartimento di Scienze e Tecnologie Biologiche, Chimiche e Farmaceutiche (STEBICEF), Università degli Studi di Palermo, 90100 Palermo, Italy

*Correspondence should be addressed to: [dario.savoca@unipa.it](mailto:dario.savoca@unipa.it); Tel: +39-3293613086

**Total SI-Figures: 7**

**Total SI-Tables: 2**

**Total SI-Pages: 6**

**Fig. S1.** The trend of the [PFOA]_CF_ for each sea urchin individual and analyzed weekly (time – day) during the experimental period in which PFOA was not added to FSW (control group).

**Fig. S2.** Histograms show the trend of the [PFOA]_CF_ for each sea urchin individual analysed weekly (time – day) during the PFOA exposure period at a theorized FSW concentration of 100 ppm

**Fig. S3.** Histograms show the trend of the [PFOA]_CF_ for each sea urchin individual analysed weekly (time – day) during the PFOA exposure period at a theorized FSW concentration of 10 ppm.

| **Individuals not exposed (0 PPM)** | **Day of death** | **[PFOA]_CF_** | **[PFOA]_G_** |
| --- | --- | --- | --- |
| X1 (M) | S (28) | 0 | 0 |
| X2 (F) | S (28) | 0 | 0 |
| X3 (M) | S (28) | 0 | 0 |
| X4 (F) | S (28) | 0 | 0 |
| Y1 (M) | S (28) | 0 | 0 |
| Y2 (F) | S (28) | 0 | 0 |
| Y3 (M) | S (28) | 0 | 0 |
| Y4 (M) | S (28) | 0 | 0 |
| Z1 (F) | S (28) | 0 | 0 |
| Z2 (F) | S (28) | 1 | 0 |
| Z3 (M) | S (28) | 0 | 0 |
| Z4 (M) | S (28) | 0 | 0 |

**Table S1.** PFOA concentration (ppb) in the coelomic fluid (CF) and gonads (G) of individuals not to pollutant at the day of death (28) by sacrificed (S). M: Male; F: Female.

| **Individuals exposed to 10 ppm** | **Day of death** | **[PFOA]_CF_**  **at day 28** | **[PFOA]_CF_ after depuration** | **% Decontamination** | **[PFOA]_G_**  **at day 28** | **[PFOA]_G_**  **after**  **depuration** |
| --- | --- | --- | --- | --- | --- | --- |
| **A1 (F)** | (S) 28 | 5.66 |  |  | 4.56 |  |
| **A2 (M)** | (S) 28+2 | 9.96 | 0.90 | 91 % |  | 6.62 |
| **A3 (F)** | (S) 28 | 0.57 |  |  | 4.41 |  |
| **A4 (U)** | (S) 28+2 | 6.99 | 0.42 | 94 % |  | n.a |
| **B1 (F)** | (S) 28+2 | 25.43 | 5.19 | 80 % |  | n.a |
| **B2 (M)** | (S) 28 | 0.41 |  |  | 40.54 |  |
| **B3 (U)** | (S) 28+2 | 2.89 | 0.09 | 97 % |  | 23.83 |
| **B4 (M)** | (S) 28 | 0.67 |  |  | 11.39 |  |
| **C1 (M)** | (S) 28 | 10.08 |  |  | 13.02 |  |
| **C2 (F)** | (S) 28+2 | 17.25 | 6.49 | 62 % |  | 16.92 |
| **C3 (U)** | (S) 28+2 | 5.43 | 0.04 | 99 % |  | 16.18 |
| **C4 (F)** | (S) 28 | 4.90 |  |  | 8.80 |  |

**Table S2.** PFOA concentration (ppm) in the coelomic fluid (CF) and gonads (G) of individuals exposed to 10 at the day of death before (7, 14, 21 or 28) or after (28+2) washout. (N): natural death; (S): sacrificed; (M): Male; (F): Female; (U): Undetermined; n.a.: not available or underdeveloped.

**
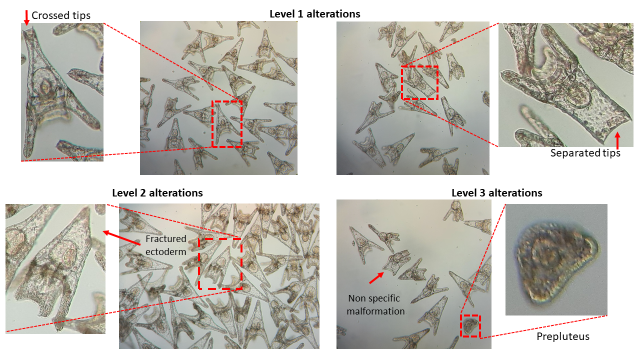
**

**Fig. S4.** Examples of presence and developmental alterations observed in larvae at the pluteus stage obtained from *P. lividus* (10x magnification).


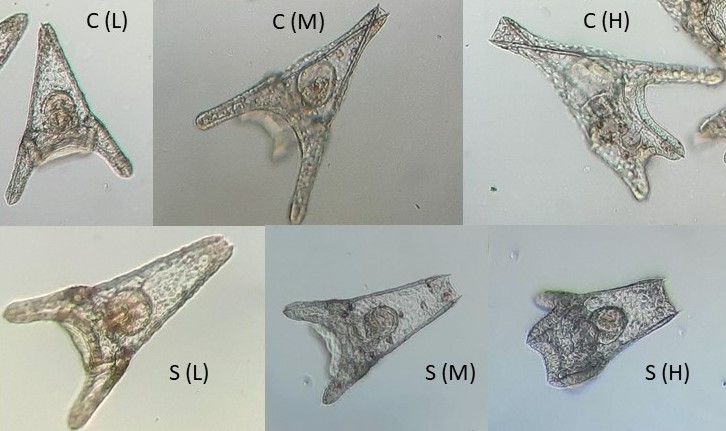


**Fig. S5.** Subcategory of level 1 anomalies with severity ranging from low grade (L) medium grade (M) to high grade (H) in embryos with crossed tips: C and separated tips: S.


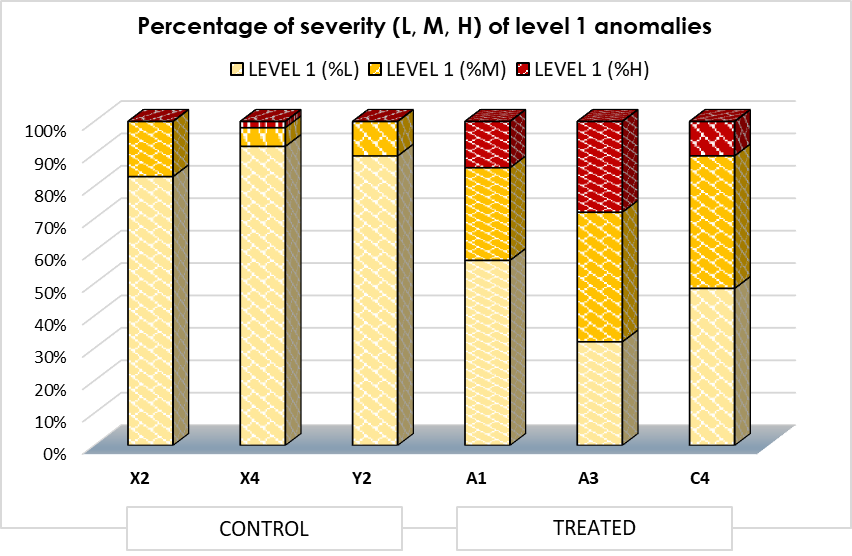


**Fig. S6.** Percentage of severity: low grade (L) medium grade (M) high grade recorded considering only level 1 malformation (100%)


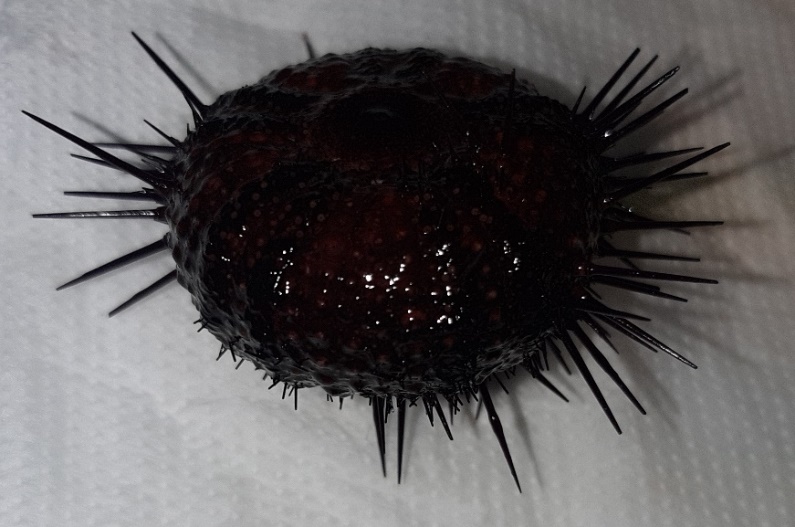


**Fig. S7.** *P. lividus* specimen exposed to PFOA showing their progressive debilitation (spines lost due to PFOA-induced stress)
